# Association of classical risk factors and coronary artery disease in type 2 diabetic patients submitted to coronary angiography

**DOI:** 10.1186/1758-5996-6-46

**Published:** 2014-03-29

**Authors:** Célia Bittencourt, Valdecira M Piveta, Carolina SV Oliveira, Felipe Crispim, Deyse Meira, Pedro Saddi-Rosa, Fernando MA Giuffrida, André F Reis

**Affiliations:** 1 Universidade Federal de São Paulo (UNIFESP)-Escola Paulista de Medicina, Diabetes Center, Rua Pedro de Toledo 910, São Paulo CEP 04039-002, SP, Brazil; 2 CEDEBA, Av. Antonio Carlos Magalhães, S/N, Salvador CEP 41820-000, BA, Brazil

**Keywords:** Type 2 diabetes mellitus, Risk factors, Coronary artery disease

## Abstract

**Background:**

Coronary artery disease (CAD) is the leading cause of death among individuals with type 2 diabetes (T2DM). T2DM accelerates atherosclerosis alongside classical risk factors such as dyslipidemia and hypertension. This study aims to investigate the association of hyperglycemia and associated risk factors with CAD in outpatients with T2DM undergoing coronary angiography.

**Methods:**

818 individuals referred to coronary angiography were evaluated for glucose disturbances. After exclusion of those with prediabetes, 347 individuals with T2DM and 94 normoglycemic controls were studied for BMI, blood pressure, fasting plasma glucose, HbA1c, lipids, HOMA, adiponectin, Framingham risk score, number of clinically significant coronary lesions (stenosis > 50%).

**Results:**

Among T2DM subjects, those with CAD (n = 237) had worse glycemic control (fasting glucose 162.3 + 69.8 vs. 143.4 + 48.9 mg/dL, p = 0.004; HbA1c 8.03 + 1.91 vs. 7.59 + 1.55%, p = 0.03), lower HDL (39.2 + 13.2 vs. 44.4 + 15.9 mg/dL, p = 0.003), and higher triglycerides (140 [106–204] vs. 121 [78.5-184.25] mg/dL, p = 0.002), reached more often therapeutic goals for LDL (63.4% vs. 51.4%, p = 0.037) and less often goals for HDL (26.6% vs. 37.3%, p = 0.04), when compared to CAD-free individuals (n = 110). The same differences were not seen in normoglycemic controls. In T2DM subjects HbA1c tertiles were associated with progressively higher number of significant coronary lesions (median number of lesions 2 [A1c < 6.8%]; 2.5 [A1c 6.8-8.2%]; 4 [A1c > 8.2%]; p = 0.01 for trend).

**Conclusions:**

Classic risk factors such as glycemic control and lipid profile were associated with presence of CAD in T2DM subjects undergoing coronary angiography. Glycemic control is progressively associated with number and extent of coronary lesions in patients with T2DM.

## Background

Cardiovascular disease (CVD) accounts for up to 80% of deaths in individuals with type 2 diabetes (T2DM) [1]. T2DM patients have a threefold higher risk than nondiabetic individuals of developing atherosclerosis and its clinical complications, such as stroke, myocardial infarction (MI), and peripheral vascular disease [2-5]. Acceleration of atherosclerosis in these patients can be due to insulin deficiency, defective insulin action, and hyperglycemia or associated metabolic defects [6]. Arterial hypertension and dyslipidemia frequently coexist with diabetes and contribute to the increased prevalence of CVD in diabetic patients [7-9]. The impact of some of these metabolic factors may be amplified by the presence of hyperglycemia. However, T2DM is regarded as an independent risk factor for cardiovascular disease [10]. The presence of hyperglycemia favors atherosclerosis and imparts an increased risk of coronary artery disease (CAD), even though the molecular mechanisms responsible for this process are largely unknown [11]. Since there are patients with T2DM that do not develop CAD, other factors besides hyperglycemia probably modulate CAD risk. Besides classical risk factors, interest has also been drawn to other measurable factors that could improve CAD risk assessment for T2DM patients, such as adiponectin and high-sensitivity C-Reactive Protein (hsCRP), among others [12]. The importance of identifying risk factors unique to CAD patients with T2DM is to control specific threats and outline goals, allowing more targeted preventive measures, thus reducing their morbidity and mortality.

Among various clinical strategies for CVD risk stratification and dynamic cardiac tests for the identification of vascular lesions, coronary angiography has a special role. Despite being an invasive procedure, it’s a gold-standard method allowing an objective analysis of the presence of atherosclerosis, its extent, and severity.

The aim of this study was to investigate the association of hyperglycemia and cardiovascular risk factors with CAD diagnosed by coronary angiography in individuals with T2DM and normoglycemia.

## Methods

A total of 818 consecutive outpatients undergoing coronary angiography at the Coronary Angiography Sector of UNIFESP University Hospital (Hospital São Paulo) have been studied. Patients were referred to the exam by their physicians for various reasons, including presence of stable angina, a positive stress test, clinical suspicion of acute coronary syndrome, or for preoperative evaluation of cardiac valvular disease or peripheral vascular disease surgeries. Clinical data, as well as personal and familial history of cardiovascular disease, diabetes mellitus and associated diseases, and medication use, were obtained by an interviewer. Patients were examined for weight, height, abdominal circumference, and blood pressure by a member of the research group. We excluded from the study patients with impaired renal function (estimated by the Modification of Diet in Renal Disease study equation, impaired renal function defined as MDRD < 50 ml/m^2^) [13], altered thyroid function, active inflammatory disease, cancer, and confirmed acute coronary syndromes. Patients with acute coronary syndromes and ST segment elevation have been excluded because thrombotic events could overestimate stenosis. Presence of T2DM was defined as previous history of diabetes mellitus (diagnosed after 40 years old) or by American Diabetes Association criteria [14]. We analyzed a subgroup of normoglycemic patients, defined by meeting all the following criteria: absence of personal history of diabetes mellitus, no use of antidiabetic medication, and no newly diagnosed diabetes (HbA1c < 5,7% and < 100 mg/dL) at recruitment, to investigate differences between individuals with or without CAD in the absence of hyperglycemia, regarding risk factors and clinical parameters. Presence of CAD was defined by any visible stenosis greater than 50% on angiography in at least one major coronary artery or branch. Arterial hypertension was defined as systolic blood pressure (SBP) ≥ 140 mmHg and/or diastolic blood pressure (DBP) ≥ 90 mmHg and/or antihypertensive medication use. Subjects were considered to have dyslipidemia if they met any of the following criteria: LDL-cholesterol ≥ 160 mg/dL, HDL-cholesterol < 40 mg/dL, triglycerides ≥ 200 mg/dL, or use of lipid lowering drugs (statins/fibrates) [15].

A blood sample was drawn after an overnight fast for analysis of fasting plasma glucose (FPG), plasma insulin, HbA1c (HPLC), lipid profile, TSH, and creatinine. The homeostasis model assessment of insulin resistance (HOMA-2IR) and β-cell function (HOMA-2B) [16] were calculated using glucose and insulin levels. We excluded from these analyses T2DM subjects using sulfonyulreas and/or insulin. Total adiponectin was measured in plasma samples using commercial ELISA kits (EZHADP-61 K, Millipore, Saint Charles, MO). Intra- and inter-assay coefficients of variation were respectively 7.4% and 10.6% (sensitivity of 0.78 ng/mL). Individuals were assessed for attaining treatment goal of major cardiovascular risk factors: blood pressure below 140 × 80 mmHg; HbA1c below 7%; LDL below 100 mg/dL (2.6 mmol/L); triglycerides below 150 mg/dL (1.7 mmol/L); HDL above 40 mg/dL (1.0 mmol/L) in men and 50 mg/dL (1.3 mmol/L) in women [14].

The Framingham score was calculated as described elsewhere [17]. In brief, gender, age, LDL-cholesterol, HDL-cholesterol, systolic and diastolic blood pressure, presence of diabetes mellitus, and current smoking status were used to calculate the 10-year risk of CAD. Individuals with calculated risk above 20% were considered as high risk for coronary events [18]. All participants gave written informed consent. The study was approved by the ethics committee of UNIFESP.

Results of continuous variables are expressed as mean ± SD for normally distributed variables and median [interquartile range] for those without normal distribution. Results of categorical variables are expressed as percentages. Comparisons of anthropometric, clinical, and laboratory phenotypes between groups were assessed by t-test and Fisher’s exact test. For all analyses, data were log transformed when the normality of distribution was rejected by the Shapiro-Wilk W test. The number of significant coronary lesions each in HbA1c tertile was analyzed by Jonckheere-Terpstra test. A p value for trend was reported in this case. Values of p < 0.05 were considered significant. Statistical analyses were performed with the SPSS 13.0 for Windows software (SPSS Inc., Chicago, IL, USA).

## Results

From a total of 818 patients who underwent coronary angiography, we identified 347 subjects with T2DM (54% male; mean age 60.8 ± 10.5 years old), from whom 92 (26.5%) were newly diagnosed. CAD was detected in 68.3% of T2DM patients. T2DM subjects with CAD (n = 237), as compared to those without CAD (n = 110), were older (61.6 vs. 59.1 years; p = 0.03) and more often male (61.2 vs. 40%; p < 0.001) and had lower BMI (28.0 ± 4.4 vs. 29.2 ± 5.3 kg/m^2^; p = 0.04). Additionally, they showed a worse metabolic profile with significantly higher HbA1c, FPG, and triglyceride levels and lower HDL-cholesterol (Table 1). T2DM patients with CAD were more frequent users of statins (72.6% vs. 60.9%; p = 0.03), even though the prevalence of dyslipidemia (~80%) was similar between groups. Presence of hypertension, 94.5% and 95.5%, was also similar between groups with and without CAD respectively. Other known classical and non-classical risk factors, such as SBP, DBP and LDL-cholesterol, renal function estimated by MDRD, and adiponectin levels were not different between groups. Diabetes duration and family history for CAD were also similar between groups (Table 1). We observed a Framingham Risk Score greater than 20% in 32.5% of patients with CAD and 21.6% in the CAD negative group (p = 0.047). No differences were noted in insulin resistance/secretion surrogates such as HOMA-2IR, abdominal circumference, and HOMA-2B.

**Table 1:** Clinical and laboratory features of patients with diabetes and normal glucose tolerance, according to the presence of CAD

	**Normoglycemic individuals**			**Diabetic individuals**		
	**Without CAD**	**With DAC**	p**	**Without CAD**	**With CAD**	p**
N	40	54		110	237	
Age (years)	55.8 (12.9)	60.2 (13.6)	NS	59.1 (10.2)	61.6 (9.8)	0.03
Male gender (%)	53.7	76.4	0.02	40	61.2	0.001
BMI (kg/m2)	27.1 (5.24)	26.4 (4.91)	NS	29.2 (5.32)	28.0 (4.40)	0.04
Abdominal circumference (cm)	95.5 (12.9)	97.0 (12.4)	NS	101.9 (12.65)	100.5 (12.00)	NS
Diabetes duration (years)*	–	–	–	4 [1-10]	6 [2-10]	NS
SBP (mmHg)	134.4 (21.8)	131.9 (22.6)	NS	145.2 (24.6)	142.3 (24.6)	NS
DBP (mmHg)	79.0 (15.4)	79.7 (14.2)	NS	82.4 (16.1)	79.2 (12.1)	NS
FPG (mg/dl)	90.4 (6.8)	90.8 (7.1)	NS	143.4 (48.9)	162.3 (69.8)	0.004
Fasting insulin (mU/L)	6.44 (4.38)	6.10 (4.77)	NS	14.6 (17.6)	10.9 (8.91)	NS
HOMA2B	69.17 (25.63)	66.50 (25.82)	NS	75.56 (51.83)	64.07 (48.51)	NS
HOMA2-IR	0.70 (0.41)	0.66 (0.42)	NS	1.84 (1.67)	1.54 (1.03)	NS
HbA1c (%)	5.28 (0.27)	5.35 (0.25)	NS	7.59 (1.55)	8.03 (1.91)	0.03
LDL (mg/dL)	105.0 (34.8)	98.6 (33.4)	NS	101.0 (39.9)	98.1 (36.2)	NS
HDL (mg/dL)	42.7 (11.4)	38.4 (10.340	NS	44.4 (15.9)	39.2 (13.2)	0.003
Triglycerides (mg/dL)*	116.0 [75.25-158.0]	115.0 [93.0-158.0]	NS	121.0 [78.5-184.25]	140.0 [106.0-204.0]	0.002
Total Adiponectin (μg/mL)*	11.80 [6.85-18.62]	8.73 [5.95-14.88]	NS	8.26 [5.49-12.86]	7.36 [5.33-11.83]	NS
eGFR (mL/min)	88.1 (22.3)	83.6 (21.4)	NS	83.1 (28.3)	80.4 (24.2)	NS
Hypertension (%)	73.2	72.7	NS	95.5	94.5	NS
Framingham risk above 20% (%)	8.6	17	NS	21.6	32.5	0.047
Statin use (%)	41.5	56.4	NS	60.9	72.6	0.03
Aspirin use (%)	58.5	67.3	NS	70	79.7	0.046
Diabetes treatment						
Insulin (%)	–	–	–	24.5	28.3	NS
Sulphonylurea (%)	–	–	–	20.4	27	NS
Metformin (%)	–	–	–	44.5	45.6	NS
Dyslipidemia (%)	70.7	89.1	0.023	89.1	90.3	NS
Familial history of CAD (%)	29.3	45.5	NS	34.9	35	NS

Values expressed in mean (SD), except where otherwise noted; *values expressed in median [interquartile range]; **p values for comparisons between individuals with and without CAD, within diabetic and normoglycemic groups.

Ninety-four normoglycemic individuals were detected in our sample (66% male; mean age 58.3 ± 13.4 years old). CAD was present in 57.4% of them. In this subgroup of patients, those with CAD were more often male (76.4% vs. 53.7%; p = 0.02) and had higher frequency of dyslipidemia (89.1% vs. 70.7%; p = 0.02) when compared to CAD-free individuals (Table 1), but both groups of normoglycemic individuals were similar in every other parameter, including use of statins and other medication (data not shown).

Regarding fulfillment of therapeutic goals for dyslipidemia and hypertension in normoglycemic individuals, no differences were observed between those with and without CAD. Among individuals with T2DM, those with CAD met therapeutic goals for LDL more frequently than those without CAD (63.4% vs. 51,4%; p = 0.04) and for HDL less frequently (26.6% vs. 37.3%; p = 0.04) (Table 2).

**Table 2:** Proportion of individuals meeting treatment goals for diabetes (HbA1c), lipids (HDL, LDL, and triglycerides), and blood pressure, according to the presence of CAD

	**Diabetic individuals**			**Normoglycemic individuals**		
	**Without CAD**	**With CAD**	**p**	**Without CAD**	**With CAD**	**p**
**N**	110	237		40	54	
**Blood pressure (%)**	26.9	36.5	NS	32.4	43.1	NS
**HbA1c (%)**	45.5	40.9	NS	–	–	
**LDL (%)**	51.4	63.4	0.037	46.2	56.9	NS
**Triglycerides (%)**	63.6	54.1	NS	72.5	73.6	NS
**HDL (%)**	37.3	26.6	0.044	35.9	28.3	NS

**Figure 1:**
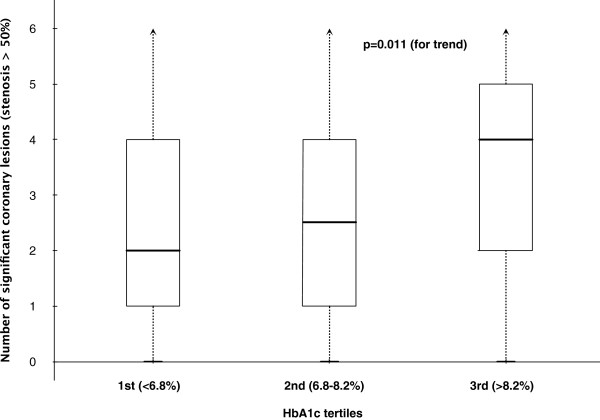
**Box-and-whisker plot of the number of significant coronary lesion (stenosis greater than 50%) per patient, according to HbA1c tertiles.**

We further stratified the 347 individuals with T2DM in FPG and HbA1c tertiles and looked at the number of significant coronary lesion (stenosis greater than 50% on angiography), as a parameter to verify the extent and severity of visible coronary disease. Median of coronary lesions were progressively higher in each HbA1c tertile (2 vs. 2.5 vs. 4; p = 0.011 for trend) (Figure 1). Similar findings were observed for FPG tertiles (data not shown). Stratification of individuals by other parameters such as triglycerides and HDL didn’t show significant association with severity or number of coronary lesions (data not shown). The same tertile analysis performed on normoglycemic individuals did not show any significant differences.

## Discussion

We have studied the clinical and laboratory characteristics of Brazilian T2DM patients with and without CAD identified by coronary angiography. In this population of T2DM subjects, some of the classical risk factors such as age, glycemic control, HDL and triglyceride levels were associated with the presence of CAD.

Glycemic control, measured by either fasting glucose or HbA1c, was associated with CAD among T2DM patients. Moreover, both glucose and HbA1c levels were associated with number of coronary lesions and severity of stenosis (Figure 1). Our results reinforce the concept that glycemic control plays a pivotal role in the presence and extension of CAD, at least in this subgroup of T2DM patients with high cardiovascular risk [6]. Even in non-diabetic individuals HbA1c can be associated with CAD and its severity [19], but this has not occurred in our sample of normoglycemic individuals. In this regard, there is some evidence from prospective studies that more intensive treatment of blood glucose in newly diagnosed T2DM patients may reduce long-term CVD rates. For instance, in the UKPDS trial, a 16% reduction in cardiovascular events in the intensive glycemic control arm was noted, but did not reach statistical significance. However, after 10 years of follow-up, patients originally randomized to intensive glycemic control showed a significant long-term reductions in MI [20], underpinning the concept of metabolic memory in this setting. Furthermore, more recent large trials [21-23] suggested no significant reduction in CVD, or even a higher risk of death, with intensive glycemic control in patients who had more advanced T2DM as compared to those on UKPDS. In the ACCORD Study, tight control did not yield protection against CVD, mainly in those with very long duration of diabetes, known history of severe hypoglycemia, and advanced atherosclerosis. Of note, all these trials were conducted in participants with more long-standing T2DM (mean duration 8–11 years) and either known CVD or multiple cardiovascular risk factors [24]. For example, the ACCORD trial involved persons who had had T2DM for a median of 10 years [21]. In our study, especially study design and sample characteristics differed markedly from ACCORD, the most striking clinical distinction being diabetes duration, roughly twice as much in ACCORD as in our sample. Moreover, the latter studied a population with several other risk factors and higher BMI.

We do not have information about the history of metabolic control of our patients particularly in the first years of disease, this period being probably important in the metabolic memory of these individuals. This fact limits a more in-depth analysis of metabolic control evolution and CAD prevalence. In our sample, diabetes duration, a well-defined risk factor for both micro and macrovascular disease [25], was not different between individuals with and without CAD. This finding could possibly derive from a hypothetically higher frequency of newly diagnosed T2DM among individuals with CAD. Nevertheless, both subgroups (with and without CAD) had a similar proportion of novel cases of T2DM (data not shown). Another possible explanation could be the relatively short median diabetes duration in our sample (~5 years). In this regard, some studies suggest that risk for CAD starts to rise significantly after about 8 years of diabetes evolution [1,25].

Dyslipidemia is a well-established risk factor for CAD in different populations, with and without diabetes mellitus [26]. In our high risk group, approximately 80% of patients had dyslipidemia. Low levels of HDL cholesterol, often associated with elevated triglyceride levels, are the most prevalent pattern of dyslipidemia in persons with T2DM [14]. Both alterations were more prevalent in T2DM with CAD in our group. This lipid modification is commonly associated with insulin resistance [27,28]. However, we found no differences between HOMA-IR between the groups. It is noteworthy that the HOMA-IR in patients with T2DM was much higher when compared to normoglycemic individuals, in agreement with the role of insulin resistance in T2DM (Table 1). If elevated LDL-cholesterol is a well defined causal risk factor for CAD, uncertainty exists about whether elevated triglyceride levels represents an additional independent CVD risk factor [25]. Of course, data from these epidemiological studies do not necessarily mean that triglycerides are not causally related to vascular risk. They could, however, be rather a marker of metabolic disturbances than a causative factor of atherosclerosis such as HDL and glycemic control [25]. We did not find any difference in LDL levels, but a higher rate of statin users in patients with CAD was seen. Besides, LDL targets for diabetic individuals could be even lower than 100 mg/dL [14]. Our data show association of worse HDL levels with CAD in diabetic individuals. However, our study design precludes any assumption of causal association. Moreover, adequately designed studies (prospective clinical trials) to evaluate the isolated role of HDL in the diabetic population are unavailable.

Hypertension, likewise, is a well-known risk factor for CAD [29]. In our study, there was no difference in frequency of hypertension and either DBP or SBP levels in diabetic individuals with or without CAD. Interestingly, we did not find association of two known cardiovascular risk factors, hypertension and elevated LDL levels, with CAD. Since the majority of our study group had both abnormalities (more than 80%), along with a high proportion of users of medication to treat these risk factor, our analyses could be unable to detect these differences.

Among T2DM individuals, there was a higher proportion of CAD individuals having Framingham risk above 20%, as compared to CAD-free individuals, with a nominally significant difference. Since our sample is composed of high risk individuals (outpatients referred to coronary angiography), this could possibly explain the small difference between subgroups. Likewise, frequencies being above the general population (20 to 30%) [18] in both groups could derive from the same fact. This could further contribute to blunt the difference between subgroups. Moreover, the validity of the score in this scenario of high risk and prevalence of overt CAD for predicting future coronary events could be questioned, since it has not been designed with this goal. Framingham risk score was not different between individuals with and without CAD in the normoglycemic group.

In the group of subjects with T2DM we observed a lower BMI in CAD positive subjects. This result should be viewed with extreme caution due to the only nominal difference and also to the fact that it was an observational study. The issue of weight and cardiovascular mortality in T2DM has been subject of intense discussion recently after the publication of the Look AHEAD study, which demonstrated that an intensive lifestyle intervention focusing on weight loss did not reduce the rate of cardiovascular events in overweight or obese adults with T2DM [30]. Weight loss may also occur from poor glycemic control, and therefore any beneficial effects of weight loss in those who intentionally lost weight are not distinguishable [31]. Recent evidence has shown that while the risk of mortality increases at higher BMIs at the time of T2DM diagnosis, there is also a higher risk of mortality for those diagnosed at a lower BMI [31,32]. Logue and Sattar pointed out that the reasons for this behavior are unknown but it may be that BMI at diagnosis reflects the underlying pathophysiology, with those diagnosed at lower BMI having either proportionately greater beta-cell failure, or else a greater diabetes predisposition via more rapid ectopic fat storage for a given BMI, factors potentially leading to worse outcomes [31]. Finally, another potential interference could stem from diabetes drugs that modulate body weight, such as insulin, sulfonylureas, and metformin. The percentage of individuals utilizing each drug, however, was not different between individuals with and without CAD, as illustrated in Table 1. Anyway, the cross-sectional nature of our data suggest that differences found in BMI could be due to selection bias rather than pathophysiologically connected to CAD.

Regarding non-classical cardiovascular risk factors, we did not find differences between adiponectin levels in diabetic patients with and without CAD. Accumulating evidence suggests that higher plasma adiponectin is a surrogate biological marker for better cardiovascular prognosis, mainly in patients with low risk of CVD [12]. In subjects with high risk of CVD, as seen in our present study group, or in those with established CAD, however, higher total adiponectin level seems to be associated with poorer prognosis [33]. A possible explanation is that higher or neutral levels of adiponectin in the CAD group may be a physiological response to limit endothelial damage in the very early stage of the atherogenic process. In more advanced disease, however, compensatory processes, including an increase in adiponectin levels, are often superseded [12].

We sought to investigate whether meeting glucose, blood pressure, and lipid therapeutic goals for adults with diabetes mellitus could have any effect on the frequency of CAD [14]. At first glance, the small percentage of individuals globally meeting the proposed goals (less than 50%) stands out. Moreover, treatment goals for cardiovascular risk factors didn’t differentiate between individuals with or without CAD in the normoglycemic group. In those with T2DM, however, an opposite behavior was seen with HDL and LDL. Less patients in the CAD-positive group met therapeutic goals for HDL, as expected. This could be due to HDL being a risk factor relatively difficult to modify. Besides, the limited efficacy of statins and fibrates in raising HDL when compared to their LDL/triglyceride-lowering capabilities could partly explain this finding. Conversely, T2DM patients with CAD met LDL treatment goals more frequently than CAD-free individuals. This is probably due to a higher frequency of statin use.

As we found no differences in major risk factors in normoglycemic subjects with or without CAD, some hypotheses can be discussed. Of note, our normoglycemic group has been selected by two combined criteria: fasting glucose and HbA1c, selecting thus individuals that are highly glucose-tolerant. One possible explanation would be that in a high risk diabetes setting, such as the one studied, risk factors are potentiated by the occurrence of diabetes/hyperglycemia. Another hypothesis would be statistical error due to the limited sample size. In this analyses, we did not aim to compare clinical parameters and risk factors between T2DM subjects and non-diabetic individuals, given the highly differing metabolic profile of both groups. Our goal was rather to assess risk factors related to the presence of CAD in a normoglycemic group, verifying if they are similar to those found in individuals with T2DM.

The major strength of our study was the use of a gold-standard method to diagnose coronary stenosis. Some limitations of our study should be discussed. Firstly, studied individuals are represented by high cardiovascular risk subjects, as they had clinical indication for cardiac catheterization. Therefore, our data are not applicable to the general T2DM population. On the other hand, this feature enhances stratification of some analyses, as, for example, in comparison with our normoglycemic group, also with a high cardiovascular risk except for hyperglycemia. Secondly, the number of subjects studied is small and results should be interpreted with caution. Moreover, OGTT were performed in an even smaller subset of individuals and could not be utilized in the analyses. Thirdly, the 50% criterion utilized to regard coronary stenosis as significant could be a potential source of bias, since thrombotic events can occur in diabetic patients in thinner but unstable plaques. Fourthly, other cardiovascular risk markers such as C-Reactive Protein have not been assessed. Finally, another important weakness of this study is its cross-sectional design, chiefly due to the cumulative effect risk factors can have with prolonged exposure and the concept of metabolic memory in the initial treatment period, both analyses being impossible in our sample.

## Conclusions

Patients with T2DM and coronary artery disease assessed by coronary angiography show higher prevalence of some non-modifiable classical risk factors such as age, but others modifiable as worse glycemic control, higher levels of triglycerides, and lower HDL. Glycemic control was associated with extension of coronary lesions and severity of stenosis in individuals with T2DM. This raises the important question whether a tighter glycemic control, or even HDL and triglycerides control for that matter, could reduce CAD rates and mortality in these patients, at least in this population displaying high cardiovascular risk but short diabetes duration comparatively to preceding studies. Naturally, this intervention must be tested in a prospective design, in populations with the same clinical and metabolic features as our sample. Further studies are necessary to evaluate coronary lesions at a functional level.

## Abbreviations

BMI: Body mass index; CAD: Coronary artery disease; CVD: Cardiovascular disease; DBP: Diastolic blood pressure; FPG: Fasting plasma glucose; HbA1c: Hemoglobin A1c; HOMA: Homeostasis model assessment; MI: Myocardial infarction; SBP: Systolic blood pressure; T2DM: Type 2 diabetes mellitus; UKPDS: United Kingdom Prospective Diabetes Study.

## Competing interests

The authors declare they have no competing interests.

## Authors’ contributions

CB, VP, FMAG, and AFR wrote the manuscript; CB, VP, CSVO, DM, and PSR researched data; FC performed laboratory measurements; FMAG performed the statistical analyses; FMAG and AFR conceived the study and supervised the project; all authors provided important input for intellectual content, having read and approved the final version.
